# Comparison of the ECOHIS and short-form P-CPQ and FIS scales

**DOI:** 10.1186/1477-7525-12-36

**Published:** 2014-03-11

**Authors:** William M Thomson, Lyndie A Foster Page, Penelope E Malden, Wanda N Gaynor, Norhasnida Nordin

**Affiliations:** 1 Sir John Walsh Research Institute, School of Dentistry, The University of Otago, Dunedin, New Zealand; 2 Capital and Coast District Health Board, Wellington, New Zealand; 3 Oral Health Department, Auckland District Health Board, Greenlane Clinical Centre, Auckland, New Zealand

**Keywords:** Oral-health-related quality of life, Children, Short-form scales, Measurement scales

## Abstract

**Background:**

The development of short-form versions of child oral–health-related quality of life (OHRQoL) scales has resulted in two closely related sets of measures. We set out to compare the properties and responsiveness of the Early Childhood Oral Health Impact Scale (ECOHIS – both “child” and “family” versions) and short-form Parental-Caregiver Perceptions Questionnaire (P-CPQ) and the Family Impact Scale (FIS) measures among New Zealand children with early childhood caries who underwent treatment under general anaesthesia (GA).

**Methods:**

Secondary analysis of data from pretest/post-test clinical studies of consecutive clinical convenience samples undertaken in Wellington in 2005 and Auckland in 2010/11, with cross-sectional analyses using the former, and longitudinal analyses using the latter.

**Results:**

Cronbach’s α values for the ECOHIS-Child, P-CPQ-16 and P-CPQ-8 were 0.80, 0.88 and 0.80 respectively, and 0.83 and 0.68 (respectively) for the FIS-8 and the ECOHIS-Family. All scales showed acceptable cross-sectional construct validity, although that of the ECOHIS-Family was not as marked as that observed with the FIS-8. Responsiveness was acceptable, with the three child-focused measures showing similar effect sizes. The two family-focused measures were also similar.

**Conclusions:**

The ECOHIS-Child and the P-CPQ scales are very similar in their properties, but the ECOHIS-Family falls short of the FIS-8 in some important ways. The ECOHIS scales may be better deployed in epidemiological survey work rather than in health services research, whereas the P-CPQ-8, P-CPQ-16 and the FIS-8 seem to be well suited for the latter (particularly with children suffering from severe caries), but their epidemiological utility remains to be demonstrated.

## Background

The last decade or so has seen considerable progress in the development, testing and validation of child measures of oral-health-related quality of life (OHRQoL). A number of competing measures now exist [1-3], with most of those intended for use with children who are old enough to give valid and reliable responses. Measuring oral health in children younger than six years of age has necessitated the use of scales which use adult informants (such as parents) who know the child well. The Parental-Caregiver Perceptions Questionnaire (P-CPQ) and the Family Impact Scale (FIS) are part of the Child Oral-Health-Related Quality of Life (COHQoL) suite of instruments developed over a decade ago [4], and are intended for use with those younger children and their families. The 33-item P-CPQ has four subscales (oral symptoms, functional limitation, emotional well-being and social well-being), and the 14-item FIS has three (parental emotions, parental/family activity, and family conflict). The validity and responsiveness of the P-CPQ and the FIS have been demonstrated recently with children undergoing dental treatment under general anaesthesia in New Zealand [5,6].

OHRQoL measures tend to be long, and short-form versions are more desirable because respondent burden is minimised and the chance of the measures being used routinely in day-to-day practice is greater. Consistent with the usual pattern with such measures, the development of short-form versions has taken two different paths and resulted in two closely related sets of measures: the 13-item Early Childhood Oral Health Impact Scale, or ECOHIS [7], and the short-form P-CPQ (both 8- and 16-item versions) and FIS (with 8 items) [8]. The ECOHIS was developed using the original 45-item pool used by Jokovic and Locker in developing the P-CPQ scales. The ECOHIS team obtained ratings of those items from health professionals (and associated staff and researchers) who were experienced in dealing with young children. The 36 items remaining from that process then underwent item reduction with a convenience sample of 30 parents of 3-5-year-old children with a range of dental care needs. This resulted in 13 items (9 child-related and 4 on family impact) which were then field-tested with a convenience sample of parents/caregivers of 5-year-olds selected for participation in a larger epidemiological study. Those children had not been selected specifically because they had early childhood caries (ECC). By contrast, the short-form P-CPQ and FIS measures [8] were developed and tested by item impact analysis of data collected in two New Zealand studies (in Wellington and Auckland) of changes in OHRQoL in ECC-affected children undergoing dental treatment under general anaesthesia [5,6]. The item impact analyses were undertaken with the Wellington study data-set, and the examination of validity and responsiveness was done with the Auckland one.

Cross-sectional construct validity, reliability and responsiveness have been shown to be acceptable for both the ECOHIS [7,9,10] and the short-form P-CPQ and FIS measures [8], but there has been no direct comparison of their properties to date. It could be argued that the similarity in item content of the ECOHIS’s child section and the 8-item short-form P-CPQ (whereby the latter contains 5 of the 9 items in the former) means that there would be little difference between them in performance. By contrast, the family section of the ECOHIS and the 8-item FIS differ considerably; the former comprises the two domains of *parent distress* and *family function*, and the latter includes the three domains of *parental emotions*, *parental/family activity* and *family conflict*, all of which were in the original FIS. Only 3 items are common to both scales, and the ECOHIS omits the item pertaining to disrupted sleep, an impact which most parents of ECC-affected children would rate as being important. Its omission most likely results from the use of an epidemiological sample (rather than a clinical one) for field-testing the ECOHIS. It might therefore be expected that the real interest in making a direct comparison of the two instruments lies in the relative performance of the components which measure the impact of a child’s condition upon the family and household. Accordingly, the aim of this study was to compare the psychometric properties and responsiveness of the ECOHIS and short-form P-CPQ and FIS scales among New Zealand children with early childhood caries who underwent treatment under general anaesthesia (GA).

## Methods

This secondary data analysis used data from pretest/post-test clinical studies of consecutive clinical convenience samples undertaken in Wellington in 2005 [5] and Auckland in 2010/11 [6]. Each study obtained prior ethical approval, and written informed consent was obtained from participants before data collection. Parents of children with ECC were asked to complete questionnaires before and after dental care provided to the child under general anaesthetic (GA). Those contained the full P-CPQ and FIS instruments (and therefore also the ECOHIS) and the global rating question “How much is your child’s overall well-being affected by the condition of his/her teeth, lips, jaws or mouth?”, scored on a 5-point scale ranging from ‘Excellent’ to ‘Poor’ (and asked before the other scales). The reference period for the baseline questionnaire was 3 months. Follow-up evaluations took place 1-3 weeks after the child’s procedure, and the follow-up questionnaire asked about the period since the child’s operation. Full details of the methods used in each study are in those earlier reports [5,6]. The IRBs were the Central Regional Ethics Committee for the Wellington sample, and the Northern X Regional Ethics Committee for the Auckland sample.

### Data analysis

Cross-sectional analyses were undertaken with the Wellington data-set, while the Auckland one was used for the longitudinal analyses, as in the previously-reported development of the P-CPQ-8, P-CPQ-16 and FIS-8 short forms [8]. Scale and subscale scores for those and the ECOHIS were computed after their internal consistency reliability was determined using Cronbach’s alpha. Cross-sectional construct validity was determined by scrutinising the gradient in means for pre-treatment scores across the global item categories of how much the child’s oral condition affected his/her overall well-being. The responsiveness of the various short forms was determined by computing change scores (through subtracting post-treatment scores from pre-treatment scores, where a positive change score represented improvement in OHRQoL), and testing the significance of the observed changes using Wilcoxon paired tests. The magnitude of change was represented by effect sizes, calculated by dividing a mean change score by the standard deviation of the pre-treatment score. Effect size statistics of less than 0.2 indicate a small clinically meaningful magnitude of change, 0.2 to 0.7 a moderate change, and greater than 0.7 a large change.

## Results

Descriptive data on the participants at baseline and follow-up are presented by sample in Table 1. There were fewer Europeans and more Pacific Island children in the Auckland sample, and it was slightly younger, on average. The follow-up rate in the Auckland sample was higher than that in the Wellington one.

**Table 1:** Number of participants in each sample at baseline and follow-up, by sociodemographic characteristics (brackets contain column percentages unless otherwise indicated)

	**Wellington**	**Auckland**
Baseline characteristics		
Number in sample	195	157
Number of females	95 (48.7)	68 (43.3)
Ethnic group		
European	70 (35.9)	28 (17.8)^a^
Māori	54 (27.7)	31 (19.7)
Pacific Island	53 (27.2)	58 (36.9)
Other	18 (9.2)	40 (25.5)
Mean age of sample (sd)	5.5 (1.5)	4.8 (1.7)^b^
Number of preschoolers	82 (42.1)	74 (47.1)
Number assessed at follow-up	124 (63.6)	144 (91.7)^c^

^a^P < 0.05; Chi-square test.

^b^P < 0.05; independent samples t-test.

^c^There were no significant sociodemographic differences between those who were followed up and those who were not.

### Cross-sectional analyses – the Wellington sample

For internal consistency reliability, Cronbach’s α values for the ECOHIS-Child, P-CPQ-16 and P-CPQ-8 were 0.80, 0.88 and 0.80 respectively. For the FIS-8 and the ECOHIS-Family, they were 0.83 and 0.68 respectively.

Data on the child-focused scales’ item content and the outcome of the item impact analyses are presented in Table 2. The three scales differ in their sampling of the original four domains: the ECOHIS-Child includes one *oral symptoms* item, four *functional limitations* items, and two each from the *emotional well-being* and *social well-being* domains; by contrast, the P-CPQ-16 and P-CPQ-8 respectively sample four and two items from each of those. The ECOHIS-Child scale includes a number of items which scored relatively low in the item impact analysis.

**Table 2:** Comparison of item content and item impact of the ECOHIS and the 16- and 8-item versions of the P-CPQ scales (Wellington sample only)

		**Short-form scale**					
**Item**	**Domain**^**a**^	**ECOHIS-child**	**P-CPQ-16**	**P-CPQ-8**	**Prevalence**^**b**^	**Mean**^**c**^	**Impact**^**d**^
Pain in the teeth, lips, jaws or mouth	OS	Included	Included	Included	66.2	2.5	166
Food caught in or between the teeth	OS		Included	Included	61.0	2.4	146
Been upset	EW	Included	Included	Included	56.9	2.5	142
Bad breath	OS		Included		53.3	2.5	133
Been irritable or frustrated	EW	Included	Included	Included	49.2	2.6	128
Difficulty biting or chewing firm foods	FL	Included	Included	Included	45.6	2.6	119
Taken longer than others to eat a meal	FL		Included	Included	44.1	2.7	119
Had trouble sleeping	FL	Included	Included		45.6	2.4	109
Breathed through the mouth	FL		Included		42.6	2.4	102
Had difficulty drinking or eating hot or cold foods	FL	Included			40.0	2.4	96
Been anxious or fearful	EW		Included		37.9	2.5	95
Acted shy or embarrassed	EW		Included		25.1	2.3	58
Missed school or preschool	SW	Included	Included	Included	23.1	2.4	55
Food stuck in the roof of the mouth	OS		Included		19.0	2.5	48
Had difficulty saying any words	FL	Included			19.0	2.4	46
Not wanted to talk to other children	SW		Included	Included	16.9	2.2	37
Had a hard time paying attention in school	SW		Included		12.8	2.6	33
Avoided smiling or laughing when around other children	SW	Included	Included		12.8	2.2	28

^a^OS = Oral Symptoms; FL = Functional Limitations; EW = Emotional Well-being; SW = Social Well-being.

^b^Percentage reporting it ‘Sometimes’, ‘Often’, or ‘Every day or almost every day’.

^c^Mean item score among those reporting it ‘Sometimes’, ‘Often’, or ‘Every day or almost every day’.

^d^The product of the prevalence multiplied by the mean score.

Data on the family-focused scales’ item content and the outcome of the item impact analyses are presented in Table 3. The two scales differ in their sampling of the original three domains: the ECOHIS-Family includes the two items with the greatest impact in the *parental emotions* domain, the third highest-impact item from the *parental/family* activity domain, and no item from the *family conflict* domain. It also includes the financial difficulties item which was not included in the original Family Impact Scale. The FIS-8 includes two *parental emotions* items, four *parental/family* items, and two items from the *family conflict* domain.

**Table 3:** Comparison of item content and item impact of the ECOHIS and the 8-item FIS, showing items ranked by impact (Wellington sample only)

**Item**	**Domain**^**a**^	**ECOHIS-family**	**FIS-8**	**Prevalence**^**b**^	**Mean**^**c**^	**Impact**^**d**^
Felt guilty	PE	Included	Included	53.8	2.7	145
Been upset	PE	Included	Included	44.6	2.5	112
Had sleep disrupted	PF		Included	41.0	2.5	103
Required more attention from you or others in the family	PF		Included	29.2	2.5	73
Taken time off work	PF	Included	Included	25.6	2.4	61
Had less time for yourself or the family	PF		Included	22.1	2.5	55
Blamed you or another person in the family	FC		Included	17.9	2.3	41
Argued with you or others in the family	FC		Included	15.9	2.5	40
Caused financial difficulties for your family	—	Included		5.1	2.2	11

^a^PE = Parental Emotions; PF = Parental/Family activity; FC = Family Conflict.

^b^Percentage reporting it ‘Sometimes’, ‘Often’, or ‘Every day or almost every day’.

^c^Mean item score among those reporting it ‘Sometimes’, ‘Often’, or ‘Every day or almost every day’.

^d^The product of the prevalence and the mean score.

**Figure 1:**
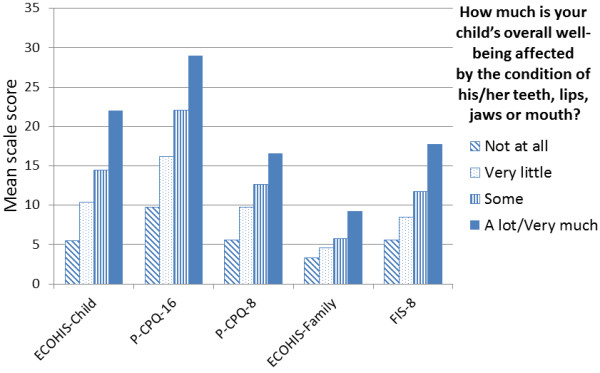
Mean scale scores by global rating of the child’s OHRQoL (Welllington sample; all score gradients were statistically significant at the P < 0.001 level).

Data depicting the scales’ cross-sectional construct validity are presented in Figure 1. All scales showed ascending gradients in their mean scores across the response categories of the global item. The gradient for the ECOHIS-Child scores was as steep as that observed with the P-CPQ-16 (and greater than that seen with the P-CPQ-8), whereas the gradient seen for the ECOHIS-Family was not as marked as that observed with the FIS-8.

### Examination of responsiveness – the Auckland sample

Data on changes associated with treatment are presented in Table 4. The three child-focused measures showed similar effect sizes. The two family-focused measures were also similar.

**Table 4:** Scale responsiveness: comparison of mean scores in the ECOHIS-Child, P-CPQ-16, P-CPQ-8, ECOHIS-Family and FIS-8 at baseline and follow-up in the Auckland sample, with effect sizes (N = 157)

	**Pre-treatment**		**Post-treatment**				
	**Mean score (sd)**	**Range (number scoring 0)**	**Mean score (sd)**	**Range (number scoring 0)**	**Mean change score (sd)**	**Effect size**	**Effect size description**
Child measures							
ECOHIS-child	7.7 (5.6)	0-28 (11)	2.6 (3.2)^a^	0-16 (52)	5.2 (5.7)	0.9	Large
P-CPQ-16	15.7 (9.9)	0-50 (3)	4.4 (5.6)^a^	0-30 (43)	11.4 (9.9)	1.2	Large
P-CPQ-8	9.0 (5.5)	0-26 (3)	2.9 (3.2)^a^	0-12 (47)	6.1 (5.5)	1.1	Large
Family measures							
ECOHIS-family	3.8 (3.2)	0-14 (26)	1.8 (2.1)^a^	0-10 (61)	2.0 (3.2)	0.6	Moderate
FIS-8	6.6 (5.6)	0-25 (18)	3.0 (3.7)^a^	0-17 (88)	3.6 (5.4)	0.6	Moderate

^a^Difference between baseline and follow-up scores significant at P < 0.0001; Wilcoxon paired test.

## Discussion

This study set out to compare the properties and responsiveness of the ECOHIS and short-form P-CPQ and FIS scales, using data obtained from the parents of New Zealand children with early childhood caries who underwent treatment under GA. It has found that the ECOHIS-Child and the P-CPQ scales are very similar in their internal consistency reliability, cross-sectional construct validity and responsiveness, but that the ECOHIS-Family and the FIS-8 differ in some important ways, despite being similar in their responsiveness.

Before discussing the findings, the limitations of the study must be considered. First, it was a secondary analysis of data collected using the long-form (original) versions of the instruments. The extracted data were used not only in the current comparison, but also in the development of the short-form P-CPQ and FIS measures. Second, ethical concerns meant that we did not investigate test-retest reliability: it would have been an imposition on parents who had already been through a stressful time. Third, we did not investigate family structure and functioning, perhaps compromising our investigation of family impact and how it changed.

Turning to the findings, it appears that, at least for determining changes in OHRQoL associated with treatment for early childhood caries, the ECOHIS-Child and the short-form P-CPQ scales are comparable. This means that investigators planning to use a parent-reported measure of child OHRQoL in monitoring the outcomes of treatment could use either measure, whether the ECOHIS or the 8- or 16-item version of the P-CPQ. However, it could be argued that, other factors being equal, it is preferable to use a measure which adequately covers all four domains. In this respect, the ECOHIS-Child’s relative oversampling of the *functional limitations* domain and under-sampling of the *oral symptoms* one may be problematic. As discussed earlier, that most likely reflects the development process for that measure, where field-testing used an epidemiological sample (rather than a clinical one), and there may not have been the same prevalence or impact of symptoms in that sample. Thus, either of the two short-form P-CPQ scales would be preferable for work in clinical samples where disease levels are high, and the ECOHIS-Child might be better deployed in epidemiological survey work.

Turning to the family impact measures, the ECOHIS-Child fell short of the FIS-8 on all aspects except responsiveness (where it was equivalent). Its internal consistency reliability was short of the 0.80 which is deemed to be acceptable [11] (although that is partly a consequence of the lower number of items). The data presented in Figure 1 indicate that its cross-sectional construct validity was inferior (if, indeed, the gradient in mean scores across the response categories for the global question can be taken to be an adequate representation of that). However, its face validity is arguably its greatest weakness, with one of the three family impact domains not sampled at all, and only one item representing another. To be fair, it does use the two *parental emotions* items which had the greatest impact, but the under-sampling of the *parental/family activity* and *family conflict* domains is problematic, and the omission of the disrupted sleep item is particularly so, given its high impact in the New Zealand data. This difference in item content clearly reflects the scales’ different provenance: that of the FIS-8 arises from its testing in children with severe caries, whereas the epidemiological origins of the ECOHIS-Family mean that the more severe effects on families were not sufficiently prevalent to make an impact.

A noteworthy inclusion in the ECOHIS-Family is the item pertaining to the child’s condition causing financial difficulty for the household. That particular item is not included in the FIS-8 because of its low impact. It placed 11^th^ out of the 14 original items, meaning that it would not have been included in the FIS-8 even if it had been seriously considered (its rank in the Auckland sample was no different from that in the Wellington sample, either). It is also worth considering that the relevance of such an item would differ according to the health system in which the instrument was used, and that would unnecessarily complicate international comparisons.

## Conclusions

This investigation of the properties and responsiveness of the ECOHIS and short-form P-CPQ and FIS scales in New Zealand children undergoing dental treatment under GA for early childhood caries has found that the ECOHIS scales have some weaknesses which undermine their suitability for use with children with that condition. They may be better deployed in epidemiological survey work rather than in health services research with high-caries-experience samples. By contrast, the P-CPQ-8, P-CPQ-16 and the FIS-8 seem to be well suited for the latter, but their epidemiological utility remains to be demonstrated.

## Competing interests

The authors declare no competing interests.

## Authors’ contributions

WMT, PEM and WNG designed the original studies; PEM and WNG conducted the studies and collected the data; WMT, NN and LFP analysed the data; WMT drafted the manuscript, and all authors critically appraised and revised the manuscript. All authors read and approved the final manuscript.
